# Deep Lossless Compression Algorithm Based on Arithmetic Coding for Power Data

**DOI:** 10.3390/s22145331

**Published:** 2022-07-16

**Authors:** Zhoujun Ma, Hong Zhu, Zhuohao He, Yue Lu, Fuyuan Song

**Affiliations:** 1 State Grid Jiangsu Electric Power Co., Ltd., Nanjing Power Supply Branch, Nanjing 210019, China; mazhoujunsgcc@163.com (Z.M.); zhuhong99@vip.sina.com (H.Z.); 2 College of Energy and Electrical Engineering, Hohai University, Nanjing 210098, China; 3 Engineering Research Center of Digital Forensics, Ministry of Education, Nanjing University of Information Science and Technology, Nanjing 210044, China; 20211220022@nuist.edu.cn (Y.L.); fysong@nuist.edu.cn (F.S.)

**Keywords:** Long Short-Term Memory, transformer, data compression, smart grid, arithmetic coding

## Abstract

Classical lossless compression algorithm highly relies on artificially designed encoding and quantification strategies for general purposes. With the rapid development of deep learning, data-driven methods based on the neural network can learn features and show better performance on specific data domains. We propose an efficient deep lossless compression algorithm, which uses arithmetic coding to quantify the network output. This scheme compares the training effects of Bi-directional Long Short-Term Memory (Bi-LSTM) and Transformers on minute-level power data that are not sparse in the time-frequency domain. The model can automatically extract features and adapt to the quantification of the probability distribution. The results of minute-level power data show that the average compression ratio (CR) is 4.06, which has a higher compression ratio than the classical entropy coding method.

## 1. Introduction

The power grid system collects huge power consumption data, which brings huge resource consumption to the communication in the transmission network. Strengthening the management and detection of the smart grid will further improve its work efficiency. Isaías González [1] proposed a multi-layer architecture with six functional layers as a new attempt at a smart grid monitoring system. The encryption of private files in any system [2,3] can further improve its supervision ability. Maik plenz [4] built a privacy framework for smart meters and increased management of smart grid security. The security of privacy files is guaranteed by the perturbation method of Gaussian distribution, and proposed two methods to encode power distribution datasets.

Different from industrial power data, household power data is collected in minutes. Tightiz and Yang [5] analyzed the characteristics and performance of the Internet of things protocol in the smart grid, which will provide a reference for the subsequent analysis of the smart grid. Huang [6] proposed an algorithm based on a depth automatic encoder, which realizes the classification and compression of power load data by extracting hierarchical features. This is helpful for further analysis of smart grid. In addition, the scheme in [7,8,9] utilized compression methods to smart grid load data. K-means singular value decomposition (K-SCD) [10] sparse representation technology is used to compress the load data of intelligent instruments.

Many data compression methods have been developed, such as the classic linear predictive coding method [11] and the discrete cosine transform (DCT) [12]. For data compression in power systems, a phasor measurement unit (PMU) data compression method based on wavelet transform is proposed in [13], and Das [14] introduced a power system data compression method based on principal component analysis (PCA). Mehra [15] proposed a compression scheme based on PCA. However, these methods did not significantly improve the compression of minute-level power data.

In addition, the calculation capacity of electricity meters in residential areas is weak. To ensure transmission efficiency, it is selected to transmit the power consumption data of multiple users per minute. This makes the data not sparse in the time domain and frequency domain. Traditional sampling methods [12,13,14] are difficult to obtain good compression results. How to efficiently compress and store these power data has become a key social problem. It is desirable to design an efficient compression algorithm to reduce the communication cost and storage overhead.

Our contributions are summarized below:

- We propose a deep lossless compression algorithm for minute level power data to compress household power data of a smart grid;
- We analyze the learning effect of networks on power data. The performance evaluation experiments of compression ratio and entropy show that deep learning will improve the coding efficiency.

We introduce the related work in Section 2. Section 3 shows the background of the deep learning network and our method is introduced in Section 4. Section 5 shows the experimental results to evaluate the performance of the scheme over power data. Finally, the conclusion and future research direction of this method are summarized in Section 6.

## 2. Related Work

Lossless compression algorithms [16,17] have been widely used. Although the compression performance is poor, and the compression speed is very superior, they do not particularly consider the nature of the power data. Power data has the characteristics of repetitiveness and slow change. The emergence of entropy coding provides a new method for lossless compression. Shannon [18] proved that the limit of compression rate is the entropy rate. Huffman coding [19] can build a tree based on the probability distribution, and the symbols with a large number of occurrences should be ahead as far as possible, but Huffman coding can not reach the entropy limit. Arithmetic coding [20] can represent a group of characters as floating-point numbers in intervals [0,1] through probability distribution, which is closest to the entropy limit.

Ringwelski [21] proposed the Huffman coding method for smart meter data compression. Das [22] proposed four arithmetic coding schemes for compressing power data. Sarkar [23,24] developed two compression algorithms based on Huffman and basic arithmetic coding for compressing combined data arrays. However, the performance of these traditional entropy coding algorithms should be improved.

The technology based on wavelet transformation has gradually matured. Khan [25] proposed a technology based on embedded zerotree wavelets (EZWT) to achieve smart grid data denoising and compression. Khan [26] developed a smart grid data denoising and compression algorithm based on Wavelet Packet Decomposition (WPD), which expanded the Wavelet Decomposition (WD) tree into a complete binary tree. Ji [27] improved the existing wavelet transform and then proposed a general data compression method for different types of signals in the power system. Cheng [28] proposed a method for compressing oscillation wide-area measurement data based on wavelet transform, which selects the wavelet function and decomposition scale according to the oscillation frequency of the power system in the wide-area measurement system. Prathibha [29] proposed a dual-tree complex wavelet transform method for power quality monitoring and combined with run-length coding technology to compress the disturbing data. Ruiz [30] used a six-level biorthogonal wavelet transform method to improve the compression rate of compressed power quality signals. However, the above methods are only suitable for sinusoidal signals, and most algorithms compress the phasor measurement unit (PMU) signal measured by the power plant.

In recent years, some new compression algorithms have been proposed. Gontijo [31] compresses power quality data with disturbances through segmentation, transformation, quantization, and entropy coding. He [32] combines cross-entropy and singular value decomposition to compress PMU signals. Karthika [33] proposed a data compression algorithm for an intelligent power distribution system based on singular value decomposition, which reduces the mean square error (MSE) after compression. Differential binary encoding algorithm (DBEA) [34] proposed a differential binary coding method with low computational load and high compression ratio. Sarkar [35] expanded the data input range on this basis, but sacrificed compression performance. Then, Sarkar [36] analyzed the performance of different types of DBEA algorithms, but DBEA can maximize performance only when there are many repetitive elements, and the effect is very poor in actual phase current and power data. Abuadbba [37] and Tripathi [38] used Gaussian approximation to improve the traditional encoding method to compress smart meter data. Experiments showed that the compression performance of the improved encoding method is significantly improved. Howeever, the algorithms of the DBEA series are suitable for having a good effect in the case of repeated data, and the effect is not good on uneven data.

Recurrent neural network (RNN) and transformer are very effective in learning time series data. They can be developed into machine translation and language modeling. Both models can learn the characteristics of data, input one character, and output the prediction probability of the next character. Arithmetic coding can be coded according to the prediction probability. The more accurate the predicted next character is, the better the compression performance of arithmetic coding.

The neural network can learn complex data. Stanford [39] combined a cyclic neural network with arithmetic coding to allocate a large probability for the possible next word and improve the performance of lossless compression. Liu [40] introduced cyclic connections for the network, retained the end of the hidden state, improved the ability of the model to capture long-term dependencies, and then used arithmetic coding to improve the compression performance. Wang [41] compressed human DNA sequences using this lossless compression method. These methods can be used for reference in power data compression.

## 3. Background

Using the measurement matrix to compress the original vector is the mainstream algorithm in the field of image compression. Although the data reconstructed by convolution neural network may have certain deviations, the success of this method is mainly due to the characteristics of human vision. However, we found that for small power consumption data, a small deviation will affect the economic benefits of the household power grid. We chose other networks to build lossless compression algorithms.

### 3.1. Bi-LSTM

RNN shows satisfying performance in natural language processing and is proved to be feasible in data compression [39]. As an improved branch of primitive RNN. Bi-LSTM has a better training effect than RNN. It controls the output of neurons through some gates, to solve the problems of gradient disappearance and gradient explosion in RNN. Its calculation formula is:

(1) $$\begin{array}{c} I_{t}=\sigma \left(W_{i}\cdot \left[h_{t-1},x_{t}\right]+b_{i}\right),\\ f_{t}=\sigma \left(W_{f}\cdot \left[h_{t-1},x_{t}\right]+b_{f}\right),\\ C_{t}=f_{t}\cdot C_{t-1}+I_{t}\cdot \tanh\left(W_{c}\cdot \left[h_{t-1},x_{t}\right]+b_{c}\right),\\ O_{t}=\sigma \left(W_{o}\cdot \left[h_{t-1},x_{t}\right]+b_{o}\right),\\ h_{t}=O_{t}\cdot \tanh\left(C_{t}\right) \end{array}$$

where *t* represents the time, *W* is the weight matrix, and $I_{t}$, $f_{t}$, $c_{t}$, $o_{t}$, $h_{t}$ are input gate, forget gate, cell state, output gate, and hidden state, respectively.

### 3.2. Transformer

Transformer, as a recently proposed network, can get the same training effect and has different advantages from LSTM in some aspects. It uses the multi-head self-attention mechanism, which will have a better effect when the training interval is long. The calculation formula is:

(2) $$\mathrm{MultiHead}\left(Q,K,V\right)=\mathrm{Concat}\left(H_{1},H_{2}\right)W^{O}$$

where $H_{1}$ and $H_{2}$ are the two self-attention modules of multi-head attention, and $W^{O}$ is the weight. The calculation formula for self-attention is:

(3) $$H_{i}=Attention\left(Q,K,V\right)=softmax\left(\frac{QK^{T}}{\sqrt{d_{k}}}\right)V$$

where *Q* is query matrix, *K* is the content that you want to pay attention to, $QK^{T}$ is the dot product operation. The purpose is to calculate the attention weight of *Q* on *V*, $\sqrt{d_{k}}$ is the scale of the dot product. Finally, the linear transformation layer and softmax layer of the decoder is output to obtain the predicted probability.

These two models can obtain similar results in the training sequence data. Although their structures are different, we can use any of them in the design of depth algorithm. Our compression algorithm is scalable and can be inserted into other training models to capture potential power features.

## 4. Proposed Method

Before building the model, we need to construct the data model. First, we cut the data, and the length of each segment can be 100, which depends on the setting of the smart grid equipment. For the power data with length n, we can model it as:

(4) $$X=\{x_{1},x_{2},\ldots ,x_{n}\}$$

where $x_{i}$ represents the electricity consumption data generated at the *i*-th moment.

**Figure 1:**
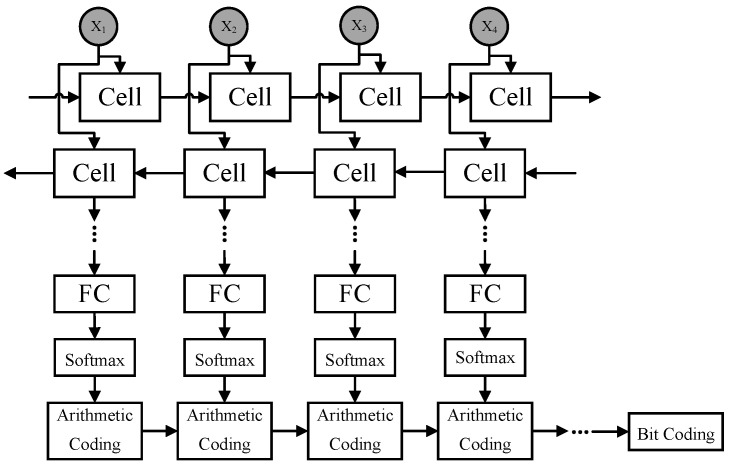
Proposed deep lossless compression algorithm framework. The upper part of the figure is the bilstm model, which includes bidirectional neurons, FC layer, and softmax layer. The lower part is Arithmetic Coding and Bit Coding.

In our proposed algorithm, the Bi-LSTM mode is used, which has significant advantages in time transmission. In the optional model, we analyzed and compared their performance. As shown in Figure 1, the algorithm framework we designed consists of two parts: model training and coding compression. The model includes bidirectional neurons, full connection layer (FC), and softmax layer. The compression part consists of arithmetic coding and bit coding. $x_{i}$ is displayed at the top of the figure as input. After processing the input with the Bi-LSTM model, the softmax layer calculates and outputs the prediction probability. Then, the prediction probability is compressed into floating-point data by arithmetic coding and finally converted into the binary stream by bit coding. We express the calculation formula of output prediction probability of the softmax layer as:

(5) $$p\left(x_{i}\right)=softmax\left(Wx_{i}+b\right)$$

where $p(x_{i})$ represents the prediction probability of $x_{i+1}$, *W* is the weight matrix, and *b* represents the deviation.

In the power data, the extraction of two-way spatial features can better capture the front and back dependencies, thereby increasing the accuracy of the prediction accuracy. In the training process, our loss function uses cross-entropy:

(6) $$L=-\frac{1}{N}\sum_{i=1}^{N}\sum_{j=1}^{M}y\left(x_{ij}\right)\log\left(p\left(x_{ij}\right)\right)$$

where $y(x_{ij})$ represents the true probability distribution, $p(x_{ij})$ represents the predicted probability distribution, *M* represents the length of the sequence, and *N* is the number of training batch samples.

**Figure 2:**
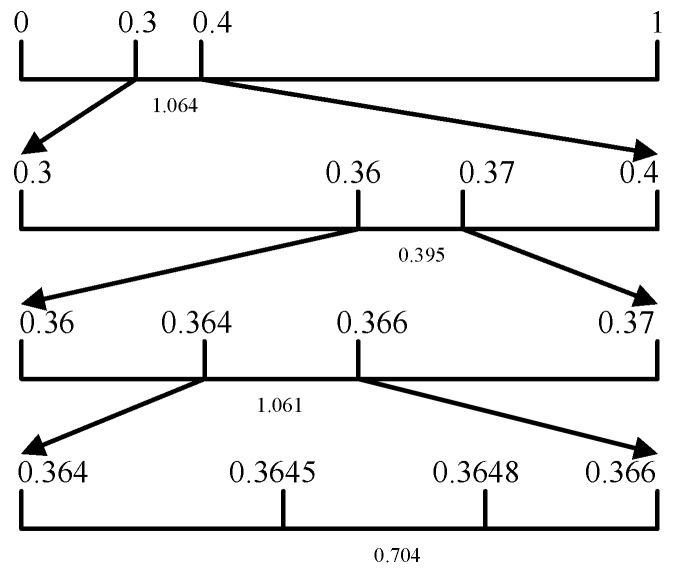
Encoding a set of data [1.064, 0.395, 1.061, 0.704] with arithmetic coding.

The compression process of arithmetic coding [18] in our framework is shown in Figure 2. Arithmetic coding compresses the output of the model, expressing the data in the interval of [0, 1], and the output are converted to floating-point type. This entropy coding is very efficient. The output probability of the model is context-adaptive, the prediction probability is closer to the source probability, the total entropy obtained is smaller, the value range of the final coding interval is wider, and the compression effect will be better than static arithmetic coding.

**Figure 3:**
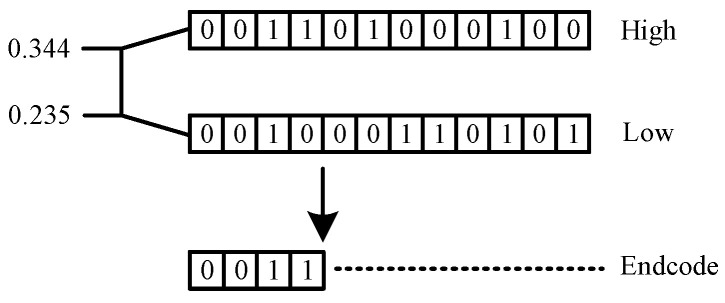
Bit coding.

As shown in Figure 3. In the last output probability interval, the left and right boundaries are converted to binary, and the final compression result is intercepted in the interval, which we call Bit encoding. The decoder is responsible for restoring the compression result to the original data. It needs the first character of the original data and the compression result as input, and it also uses iteration to restore the original data. Among them, the distribution of probability determines the entropy, increases the utilization efficiency of the interception section, and plays a role in determining the compression performance. Adaptive arithmetic coding can obtain a coding interval closer to perfect.

We use the compression ratio given in Equation (7) as an evaluation criterion to compare the performance of our method with other methods:

(7) $$CR=\frac{originaldata(inbytes)}{encodedata(inbytes)\;}$$

Entropy, like the compression ratio, is also an important parameter of the compression algorithm, which is the expected value of the information contained in each message. In some cases, it can reflect the compression performance of the algorithm [36] for the cutting segment *X* containing different *N* characters. We use entropy as another performance metric, and the calculation formula is:

(8) $$P\left(X\right)=-\sum_{i=1}^{N}p\left(x_{i}\right)\log_{b}p\left(x_{i}\right)$$

where $p(x_{i})$ is the prediction probability, *N* is the number of different characters, $x_{i}$ is the selected data, and and the base number b=2.

## 5. Experimental Results

In this section, to better test the impact of the model on compression performance, we use a private dataset, which contains user electricity data collected by a power supply company in different stations. When training the data, our environment is deployed in GeForce GTX 1080 Ti, and we adjust the parameters of the model to find a suitable threshold between model accuracy and size, making the algorithm more suitable for the real scene. We set the hidden layer feature to 32, and finally extract 16 features in the fully connected layer. This also achieve the simplification of the model, so that the algorithm can be deployed in the central processing unit (CPU) environment and ensure that the accuracy of the model is guaranteed. In the real scenario, the collection meter is deployed in the living area of users, and there is not too high a configuration. Therefore, a lightweight model is required.

We improve the calculation speed of the model by controlling the parameters of the hidden layer. It takes about 2 h to train on a single graphics processing unit (GPU). In the real scene test, we can achieve the average time of a single call to the model less than 0.1 s on the CPU; unfortunately, however, as the length of compressed data increases, the overall time consumed is still very long.

In the Bi-LSTM model, we use one-hot to encode the character sequence into a K-dimensional vector sequence and use it as the input of the network. The mode output is the probability distribution of the next character. We use the cross-entropy function to minimize loss. The state of the neural unit is retained across batches, and the Adam optimizer is used to solve the gradient explosion problem in the model. The Bi-LSTM model settings are shown in Table 1.

**Figure 4:**
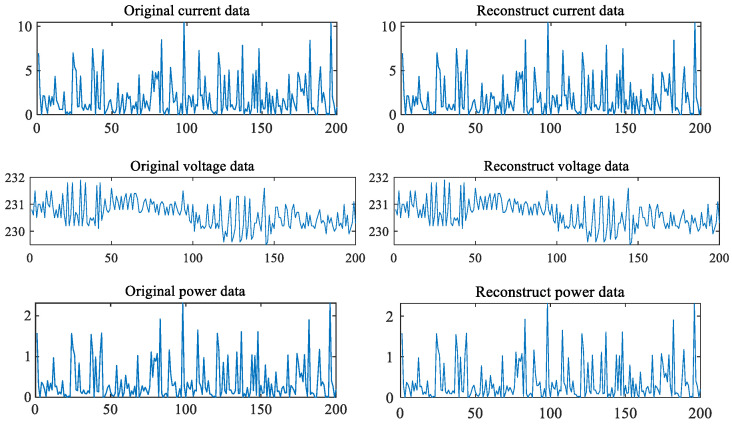
Original data and reconstructed data.

Figure 4 shows the experimental effect of our proposed method on power data. Our data is the actual power consumption value of users. The data is provided by a power supply company. The data is collected in String format. The experiment tests the power consumption data of 200 users in the same minute. The abscissa is the user and the ordinate is the value.

Most of the traditional compression algorithms do not learn the complex characteristics of the data and are not good at modeling the actual electricity consumption data. As shown in Table 2, we have tested two models to model the character dataset. Among them, the effect of the Bi-LSTM model is slightly better than that of the Transformer. The self-attention of the Transformer usually has a larger weight on the data at a short distance and a smaller weight at a long distance. At this time, the effect of relying solely on attention to establish a dependency relationship is not as good as that of the Bi-LSTM. It has the same situation with static arithmetic coding and Huffman. The more different elements the data has, the higher the accuracy of the output probability, resulting in a higher compression rate of the final calculation. The compression ratio of current and voltage is significantly higher than that of voltage.

Considering that the user’s actual electricity consumption data is non-sinusoidal, the wavelet transform fails here. We use traditional lossless compression methods to compare with our algorithm. Table 3 shows our comparison results with other methods. The experimental results show that our model performance is better than static arithmetic coding and the Huffman algorithm. The output of the model is closer to the true value, which can effectively save the accuracy of probability. It shows that our predicted data is close to the actual results, and the predicted probability distribution is more suitable for compression, which further proves the superiority of the algorithm.

As shown in Table 4, we show the entropy before and after compression in different algorithms in the test. The implementation shows a set of 200-min test results, and the entropy before compression is higher than that after compression. When the length of the output string is large and the repetition is not high, the larger the entropy after compression will be. This means that the greater the difference between them, the better the compression effect. The results show that the entropy of our proposed algorithm after compression is lower than that of the classical algorithm. We analyzed the positive effect of the deep learning model on entropy. The output probability of traditional coding is fixed, while the prediction probability of Bi-LSTM and Transformer models will change adaptively after training. The output is closer to the real value, which will save memory for subsequent encoding and compression. The training results of the Bi-LSTM model are slightly better than that of Transformer. We concluded that the probability of adaptive change will improve the CR and entropy, which confirms that the training of the deep learning model will have a positive impact on them.

## 6. Conclusions

In this paper, we use a deep neural network with arithmetic coding to learn the characteristics of power data. The proposed scheme realizes efficient deep lossless compression of power data and compares the improvement of arithmetic coding compression performance of two different deep learning model training power datasets. After adjusting the model parameters, our algorithm still achieves better results. In our future work, we will improve the model and further improve the compression performance and speed.

## Figures and Tables

**Table 1:** In the model setting, the input length is set to 50, the output length is 7820, and the batch is set to 128.

Layers	Process	Output Size
Layer 1	Embedding	[128, 50, 50]
Layer 2	Bi-LSTM	[128, 50, 32]
Layer 3	Bi-LSTM	[128, 32]
Layer 4	FC	[128, 16]
Layer 5	FC	[128, 7820]

**Table 2:** Compare the CR results of using Bi-LSTM and Transformer.

Model	Bi-LSTM			Transformer		
	Voltage	Current	Power	Voltage	Current	Power
Original size(in bytes)	922	888	1007	922	888	1007
Compressed size(in bytes)	174	276	304	167	296	317
CR	5.30	3.22	3.31	5.52	3.00	3.18

**Table 3:** Comparison of different compression algorithms (avg).

Algorithm	Ours	AC [24]	Huffman [23]
CR	4.06	3.31	1.98

**Table 4:** Test the entropy of a group of 200-min current data before and after compression in different algorithms.

Algorithm	AC [24]	Huffman [23]	Ours (Bi-LSTM)	Ours (Transformer)
Entropy before compression	3.23	3.23	3.23	3.23
Entropy after compression	2.73	3.14	2.17	2.24

## Data Availability

Not applicable.

## Abbreviations

The following abbreviations are used in this manuscript:

PMU	Phasor Measurement Unit
MSE	Mean Square Error
RNN	Recurrent Neural Network
LSTM	Long Short-Term Memory
Bi-LSTM	Bi-directional Long Short-Term Memory
CR	Compression Ratio
CPU	Central Processing Unit
PCA	Principal Component Analysis
DBEA	Differential Binary Encoding Algorithm
